# Advancing the Design of High‐Efficiency Printable Hole‐Conductor‐Free Mesoscopic Perovskite Solar Cells Through Machine Learning

**DOI:** 10.1002/advs.76258

**Published:** 2026-06-22

**Authors:** Hao Meng, Jingzi Zhang, Xu Zhu, Yuelin Wang, Antai Yang, Kailong Hu, Chengquan Zhong, Jiakai Liu, Menghan Dun, Xi Lin

**Affiliations:** ^1^ School of Materials Science and Engineering Harbin Institute of Technology Shenzhen China; ^2^ Research Institute of Physical Sciences in Special Environments Harbin Institute of Technology Shenzhen China; ^3^ Institute of Renewable Energy and New Quality Productive Forces Jimei University Xiamen China; ^4^ Research Center for Energy and Chemical Engineering Xinjiang Technical Institute of Physics & Chemistry Chinese Academy of Sciences Urumqi China; ^5^ University of Chinese Academy of Sciences Beijing China

**Keywords:** machine learning, perovskite solar cells, power conversion efficiency, printable mesoscopic perovskite solar cells

## Abstract

Breaking through the power conversion efficiency (PCE) limits of printable mesoscopic perovskite solar cells (p‐MPSCs) with machine learning (ML) shows great potential, but has not yet been accomplished. This work establishes a reliable workflow by constructing a high‐quality p‐MPSCs database for ML model development, followed by strategy formulation for achieving high‐performance p‐MPSCs. In the 8 validation experiments, the stacking ML model demonstrates excellent performance, with the prediction error not exceeding 2.16%. Model interpretability analysis reveals key factors influencing device performance and enables the formulation of screening rules for high‐quality precursor additives based on molecular fingerprinting. This validated framework guides the experimental realization of p‐MPSCs with a notable PCE of 19.36%, while theoretical projections suggest a maximum achievable efficiency of 24.32% through optimized design space exploration. A novel paradigm for accelerated discovery of p‐MPSCs is established through the synergistic integration of interpretable ML models and targeted experimental validation.

## Graphical abstract

Based on the largest printable mesoscopic perovskite solar cells database we established, stacking model achieved precise PCE prediction (*R* ^2^ = 0.73, MAE = 2.18%). Multiple experiments verified the accuracy of the model, which guided the fabrication of high‐PCE devices with an efficiency of 19.36%. Optimization strategies revealed a maximum attainable efficiency of 24.32%, providing key insights for advancing printable mesoscopic perovskite solar cells.

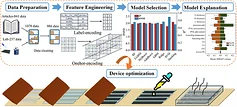

## 1 Introduction

Printable mesoscopic perovskite solar cells (p‐MPSCs) featuring mesoporous TiO_2_ (mp‐TiO_2_)/mesoporous ZrO_2_ (mp‐ZrO_2_)/porous carbon architectures stand out for elimination of hole transport layers and precious metal electrodes [1, 2, 3, 4, 5]. Nevertheless, the record power conversion efficiency (PCE) remained at 23.2% for more than 10 months (Table S1), while further improvements are required to approach the theoretical PCE limit of over 30% [6, 7, 8, 9, 10, 11, 12]. Given the multitude of factors affecting the fabrication process of perovskite solar cells (PSCs) and the complex nonlinear dependencies involved, traditional trial‐and‐error experimental approaches face fundamental challenges in addressing these coupled mechanisms [13]. In contrast, machine learning (ML) techniques exhibit transformative potential by enabling rapid analysis of multidimensional data and identifying the key determinants of target properties [14, 15, 16, 17, 18, 19, 20]. In our previous research, we developed an ML framework that integrates key parameters, including device architecture, perovskite composition, and fabrication processes. This approach enabled comprehensive optimization of PSCs. Concurrently, we designed a multimodal convolutional neural network to extract microstructural features from scanning electron microscopy (SEM) images of perovskite films. By incorporating microstructural image data into the ML model, we further enhanced the design and performance of high‐efficiency PSCs [21]. Based on these findings, we propose a novel workflow for the fabrication of highly efficient p‐MPSCs, that can integrate process optimization, molecular fingerprint screening, and targeted optimization from multiple perspectives to enable high‐efficiency strategy development [22].

**FIGURE 1:**
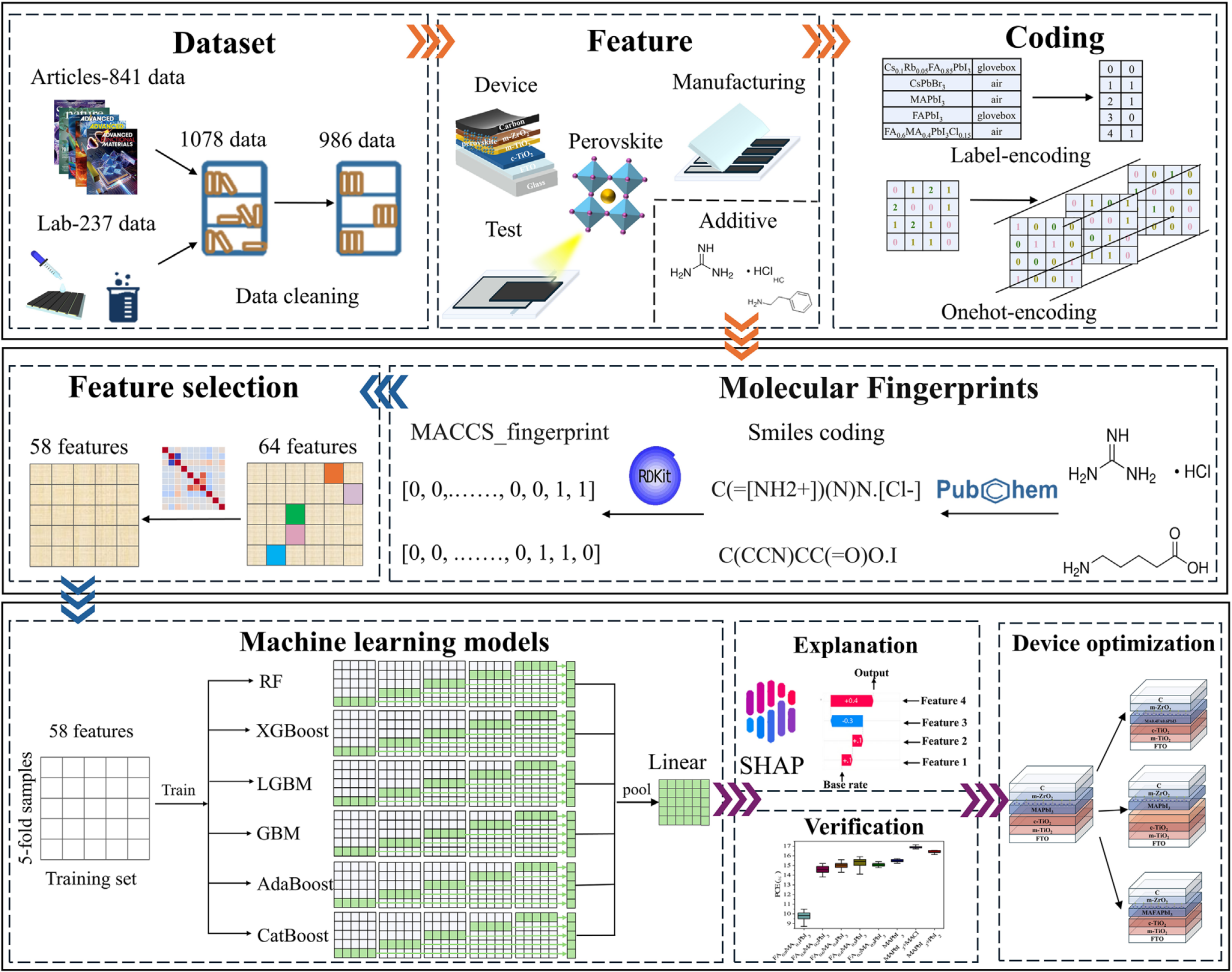
Workflow of the stacking ML model for accurate PCE prediction and discovery of potential new p‐MPSCs. The approach involves developing a stacking ML model and conducting interpretability analysis, enabling the rapid screening of optimization strategies and facilitating the fabrication of high‐performance p‐MPSCs.

In this work, the ML‐accelerated framework was developed to systematically analyze the relationship between preparation conditions and performance in p‐MPSCs. As illustrated in Figure 1, a curated database of high‐quality p‐MPSCs was constructed using 237 in‐house experimental records and 841 entries from literature published over the past five years. Based on predefined data cleaning rules, we conducted a preliminary screening and removed 92 entries from the dataset. Subsequently, the dataset was processed (including missing value handling, standardization, and normalization) to obtain a complete final version. The corresponding procedures and rules are provided in the Supporting Information and illustrated in Figure S1. A stacking ML model was developed to predict the PCE of p‐MPSCs and ensured the high quality of the model on limited datasets. Interpretability analysis of the ML model was employed to identify key factors influencing the PCE of p‐MPSCs. Considering that precursor addition is one of the most important features affecting PCE in the model training results, 231 databases of precursor additives were established for the molecular access system (MACCS) fingerprint. With the aid of ML model analysis, we established screening rules for potential high‐performance precursor additives based on MACCS fingerprint for the discovery of new materials for p‐MPSCs devices. By extracting features related to device fabrication processes and structural characteristics, the ML model provided comprehensive and actionable guidance for experimental optimization [23]. Based on the optimization strategies proposed by the ML model, the fabrication process of p‐MPSCs was refined, resulting in a significant improvement in PCE to 19.36%. Potential pathways for further enhancing device performance were also outlined, offering insights to support future breakthroughs in p‐MPSCs efficiency.

## 2 Results and Discussion

### 2.1 Prediction of PCE and Experimental Validations

Through laboratory experiments and the collection of literature data from 2018 to the present, a dataset consisting of 1,078 entries related to the fabrication of p‐MPSCs via screen printing was established. Using the method described in Table S2, a total of 15 process‐related factors (ETL, Spacer, Functional layer, perovskite components, precursor_solution, deposition procedure, deposition method, solution_add, precursor_add, type, pretreatment, Annealing, thickness, add‐Cl, add‐Pb) were selected. For instance, from the perovskite composition factors, we extracted eight ion ratio features, including methylammonium (MA^+^), formamidinium (FA^+^), Cs^+^, Rb^+^, Pb^2+^, Sn^2+^, Br^−^, and I^−^, and predicted bandgap characteristics using the model developed by Gok et al. [24] illustrated in Figure S2. The remaining factors were encoded as described in Table S2, resulting in a final dataset consisting of 54 features.

To enhance the predictive accuracy of the model, we employed a stacking ensemble learning algorithm to predict the PCE. Random Forest (RF) [25], Light Gradient Boosting Machine (LGBM) [26], Gradient Boosting Machine (GBM) [27], Adaptive Boosting (AdaBoost) [28], Extreme Gradient Boosting (XGBoost) [29], and Categorical Boosting (CatBoost) [30] were selected as base learners, while a Linear Regression (LR) [31] model was used as the meta‐learner. The lab data and article data were respectively randomly divided into a training set of 80% and a test set of 20% in a single instance and then integrated to ensure that they were evenly distributed in both the training set and the test set. To mitigate the risk of overfitting, five‐fold cross‐validation was employed during the training of the base models. Furthermore, to prove the superiority of the stacking model, we trained the six base models separately at the same time. The combination of grid search and genetic algorithm is used to optimize the hyperparameters, and optimization was performed strictly within the training folds during cross‐validation. The predictive performance and accuracy of the stacking model were comprehensively evaluated using multiple metrics, including the Pearson correlation coefficient (r), coefficient of determination (*R* ^2^), root mean square error (RMSE), and mean absolute error (MAE).

**FIGURE 2:**
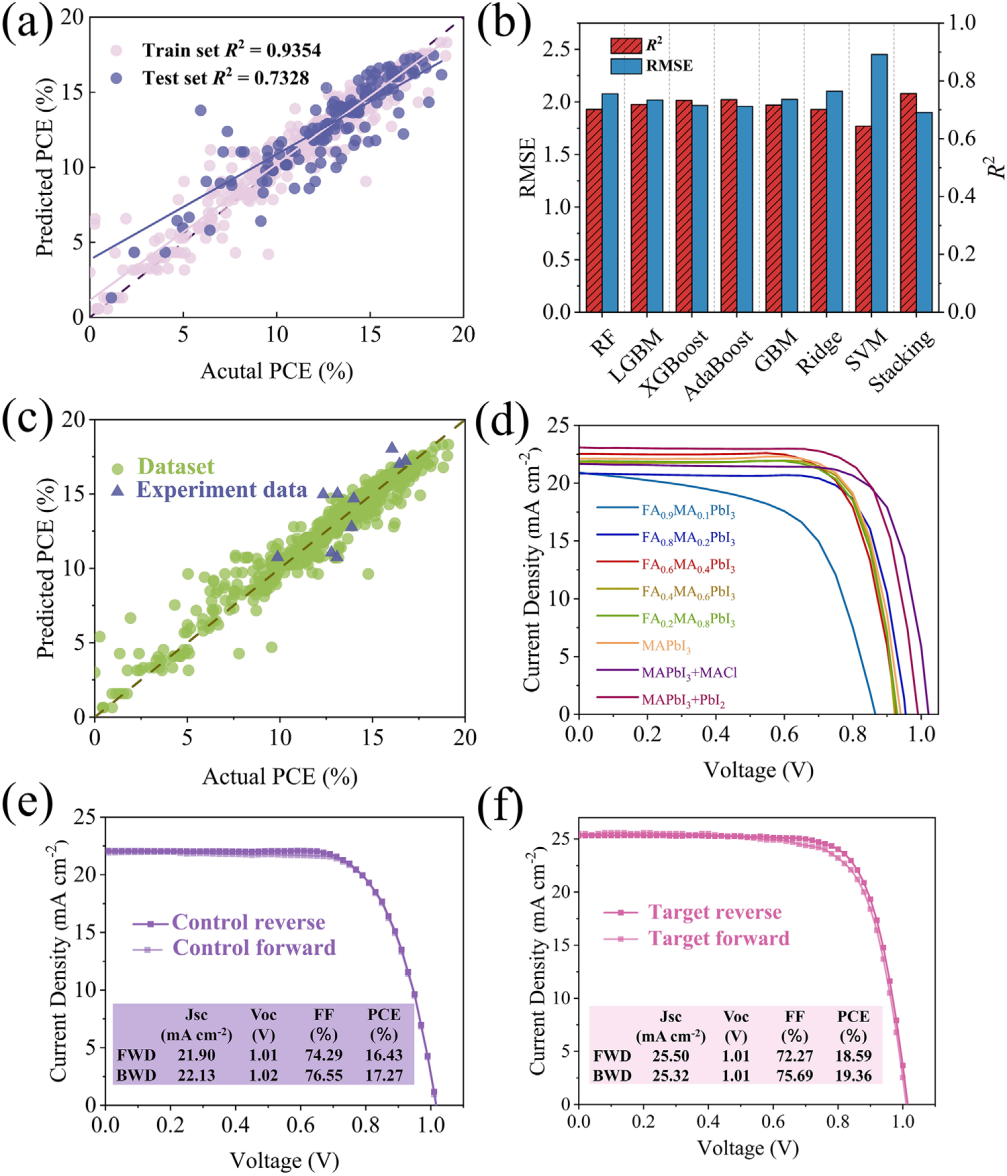
(a) The fitting graph by the stacking model. (b) The RMSE and *R* ^2^ effects of each single model and stacking model on the database. (c) The distribution of experimental results and data points from the database in the fitting graph. (d) *J*‐*V* curves of devices with different perovskite components. (e) *J*‐*V* curves of control devices and (f) *J*‐*V* curves of target devices that were optimized by the strategies provided by the ML model. Among them, FWD refers to the forward scan curve of the device, and BWD refers to the backward scan curve of the device.

The fitted scatter plots for the training and testing datasets generated by different ML models can be found in Figure S3. All models demonstrate positive fitting performance on the high‐quality dataset we constructed, achieving relatively high evaluation scores across the board, with the best hyperparameters for different models listed in Table S3. Among them, the stacking model achieved the highest performance on the test set, as shown in Figure 2a. The regression indicators of the stacking model and each model are illustrated in Figure 2b. Notably, the stacking model outperforms the other models in all aspects, owing to its ability to leverage the complementary strengths of multiple heterogeneous base models, as shown in Figure S4. Among the ML models tested, the stacking model achieved the highest *R* ^2^ of 0.73, the highest r of 0.85, the lowest RMSE of 1.75%, and the lowest MAE of 2.18%. Furthermore, we conducted paired t‐tests and analysis of variance. The corresponding results are presented in Figure S5 and Table S4. The t‐stat values of the stacking model were larger compared to the other base models, demonstrating a significant gap in error between the two, and the p‐value was less than the significance level (0.05). It is proven that the stacking model demonstrates significant advantages in multiple base learners, demonstrating the superiority of the stacking model. According to Table S5, our scheme has achieved high prediction performance in the PCE prediction task with a large amount of data and filled the research gap of ML in predicting the PCE of high‐performance p‐MPSCs devices. To validate the accuracy of the model, eight groups p‐MPSCs devices with different perovskite compositions (precursor solution: DMF/DMSO 4:1) were fabricated as shown in Table S6. The alignment of the experimental data with the regression curve and the *J*‐*V* curves of the fabricated devices are illustrated in Figure 2c,d respectively. Based on the results shown in Figure S6 and Table S6, we can observe that the experimental results closely match the model predictions, with deviations between the experimental and predicted values not exceeding 3% across all eight sets. This demonstrates the robust generalization capability of the model.

To maximize the device's PCE, a greedy algorithm combined with iterative local optimization was employed to systematically identify and prioritize the most promising solutions for refining the remaining experimental parameters. A representative device from our initial dataset was selected for detailed optimization. Drawing on the work of Zhang et al., where the inclusion of PACl as a perovskite precursor additive yielded an impressive short‐circuit current density (*J*_sc_) of 24.13 mA/cm^2^ and a PCE of 18.06%, we use PACl as an additive, following the methodology outlined in the reference [32] named Control. Using the optimized key parameters given by the ML model, based on the control devices, we added a small amount of MACl to adjust the Cl ion content to the optimal value, and 5% formamide was added to the precursor solution to promote uniform and stable solvent evaporation during film formation, following the methodology described in reference [33] and named Target.

The experimental results confirmed the effectiveness of the ML‐guided optimization strategy. The PCE of the perovskite solar cell was improved from 17.27% for the control device (Figure 2e) to 19.36% for the optimized device (Figure 2f). This enhancement was accompanied by significant improvements in other photovoltaic parameters, including an increase *J*_sc_ from 22.13 to 25.32 mA/cm^2^, while the open‐circuit voltage (*V*_oc_) and fill factor (FF) remained consistent. More experimental details are shown in Tables S7 and S8. The observed improvements are in good agreement with the phenomena predicted by the ML model, validating the rationale and effectiveness of the high‐efficiency strategy proposed by the model.

### 2.2 Model Explanation

**FIGURE 3:**
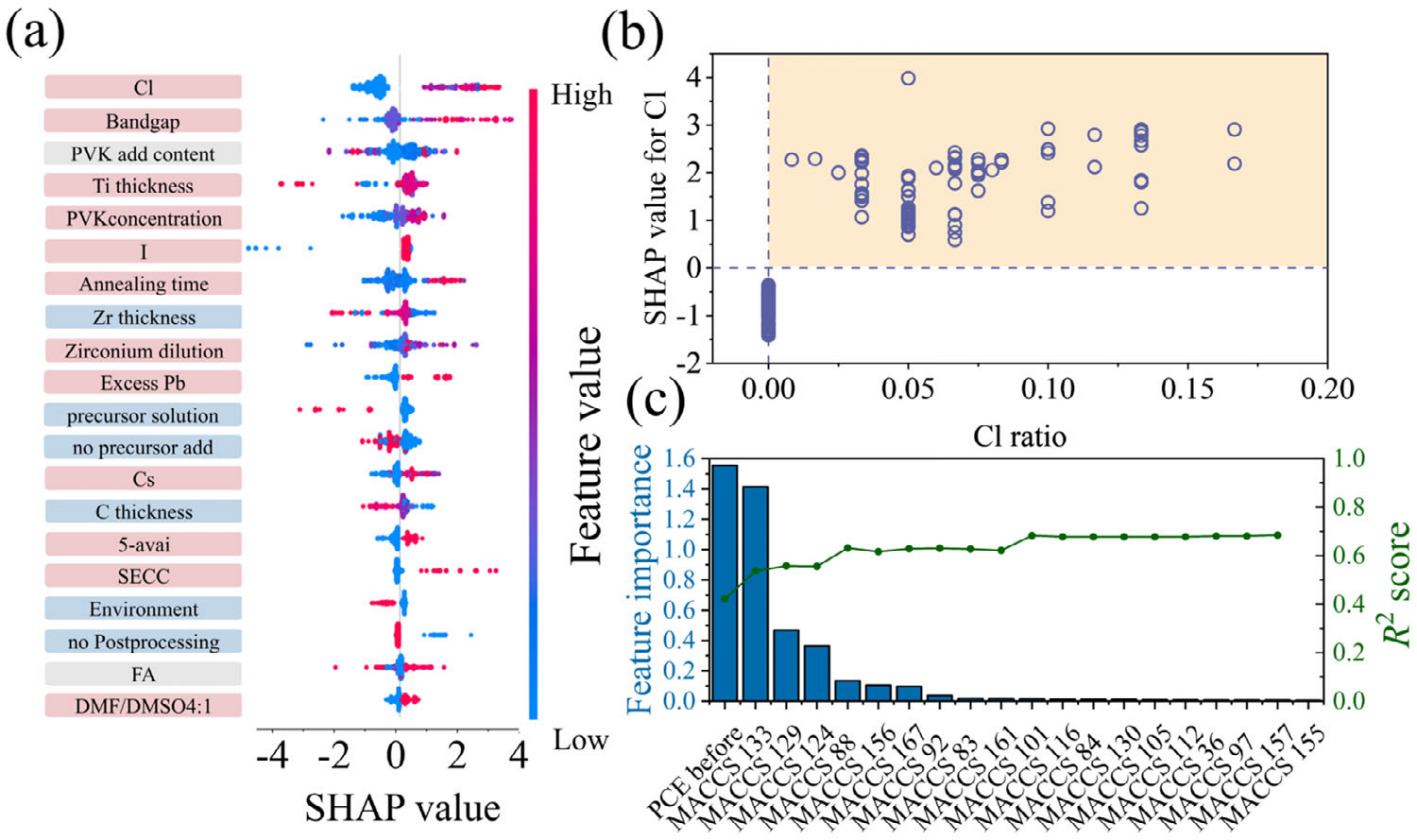
(a) SHAP values for different characteristics, sorted by importance of features. (b) The SHAP value of the Cl cation ratio to PCE. (c) The importance of the additive MACCS fingerprint feature and the *R* ^2^ applied to the model when the feature is learned.

SHapley Additive exPlanations (SHAP) were employed to analyze the model and determine the significance of various features [34]. The contribution of each feature to the PCE was analyzed using the stacking model, and a SHAP value bee swarm plot was generated to facilitate the interpretation of feature importance, as shown in Figure 3a. The horizontal axis represents the values of the respective features, where blue dots indicate a negative impact on the device PCE, and red dots denote a positive impact. The influence of each feature on PCE, whether positive or negative, was determined based on its corresponding SHAP values. It is important to note that the SHAP analysis presented herein quantifies feature contributions to the model's predictive output. While these insights provide heuristic guidance for experimental optimization, they do not substitute for first‐principles causal analysis. The analysis reveals that specific material parameters exert predominant influences on PCE, with particular characteristics demonstrating critical dominance in device optimization. From the perspective of model feature importance, Cl incorporation is identified as the most discriminative statistical predictor for PCE variation within our dataset, followed sequentially by perovskite bandgap modulation and precursor additive engineering.

As evidenced by the SHAP analysis in Figure 3b, Cl integration induces a substantial increase in feature importance metrics. This observation is consistent with established performance benchmarks. A systematic review confirms that state‐of‐the‐art p‐MPSCs universally utilize chlorine‐containing precursor formulations to achieve record efficiencies, thereby establishing a clear correlation between the incorporation of Cl integration and the optimization of device performance [35, 36, 37]. As the second major influencing factor on PCE of the device, the selection of bandgap is of utmost importance for its effects on the absorption range of light by the device [38, 39, 40, 41]. This precisely corresponds to the two research directions of p‐MPSCs devices: the MAPbI_3_ system with NMF solution as the solvent and the FA, Cs doped perovskite system with DMF/DMSO mixed solution as the solvent. Consistent with our SHAP plot, both the MA‐based perovskite with a wide bandgap and the FA, Cs doped perovskite devices with a narrow bandgap have achieved high PCE. In order to get a more intuitive understanding of the relationship between feature values and PCE, the distribution map is shown in Figures S7 and S8. In particular, in our characteristic “environment”, we found that the annealing atmosphere in the glove box (i.e., a constant temperature of 25°C and a humidity maintained at 30%) tended to achieve higher PCE compared to annealing in the air. This might be because this atmosphere provided more favorable conditions for perovskite crystallization, including stable heat transfer inside and outside the cavity and suitable water and oxygen content for perovskite crystallization. Meanwhile, the emerging annealing method SECC will also achieve considerable PCE. Meanwhile, additional SHAP analysis of emphasized features, such as precursor additives, precursor solutions, and so on, is presented in Figures S9 and S10. Specifically, based on the SHAP analysis, the optimal precursor solvent composition feature descriptor was identified as DMF/DMSO in a 4:1 ratio, while the most favorable precursor additives feature descriptor included MACl, 5‐avai, and other selected additives. Furthermore, the optimal precursor solution additives feature descriptors were determined to be FA and DMSO. Earlier research focused more on analyzing the process, but the parameters related to the device structure still remained ambiguous. To further address this issue, based on our dataset analysis, we have clarified the relevant high SHAP value parameter range, namely, the ideal thicknesses for the TiO_2_ layer, ZrO_2_ layer, and carbon layer were found to be 700 nm, 2 µm, and 15 µm, respectively.

Perovskite precursor additives, ranking third in significance, play a crucial role due to their substantial impact on the quality of perovskite crystallization, resulting in a critical selection [42, 43, 44]. Given the vast number of potential additive molecules, screening high‐quality precursor additives remains challenging. Therefore, using the RDKit cheminformatics toolkit, we calculated molecular fingerprint descriptors for 231 p‐MPSCs precursor additives in the database. Figure 3c visualizes the importance of the top 20 features and illustrates their change relative to the cumulative *R* ^2^ value, and the SHAP analysis is shown in Figure S11. Notably, after the accumulation of the tenth feature, the model's cumulative *R* ^2^ begins to stabilize, with subsequent features contributing minimal improvement to the model's performance. This suggests that these features encapsulate most of the effective information. K‐fold cross‐validation was introduced to avoid the problem of model overfitting, and we identified the 10 most influential MACCS fingerprints exhibiting the strongest correlation with device efficiency. Subsequent SHAP value analysis revealed that specific fingerprint patterns, particularly MACCS 133 and MACCS 88 demonstrate significant positive contributions to photovoltaic performance, suggesting that such structures may serve as favorable molecular features for precursor additive design.

Among them, the MACCS133 fingerprint has the highest tendency to enhance device performance. MACCS133 fingerprint (SMARTS mode *@*! @[#7] or its simplified form A$A! N) describes a nitrogen atom (N) passing through a non‐cyclic bond (!). The structural characteristics of atoms connected to a ring system. The mesoporous structure of p‐MPSCs makes perovskite crystallization more complex and prone to a large number of defects (such as lead vacancies, iodine vacancies, and other uncoordinated Pb^2+^). These defects will become non‐radiative recombination centers of carriers, reducing solar cells’ performance. The nitrogen atom (N) in the additive molecules with MACCS133 characteristics usually has a lone pair of electrons and can act as a Lewis base, undergoing strong coordination interactions with the uncoordinated Pb^2+^ (Lewis acid) in perovskite. This effectively passivates lead‐related defects, suppresses non‐radiative recombination, and thereby increases *V*_oc_ and FF. Some molecules with similar structures, such as EHA, have been proven to be effective means to increase the voltage and efficiency of devices. Further, some chemical substances with similar structures, such as nicotine and pyridine‐N‐oxide, which have not been developed as additives for p‐MPSCs, have been screened out based on our molecular fingerprint rules. The IDs of the selected MACCS molecular fingerprint and its corresponding SMART Code, and the remark are shown in Table 1, meanwhile, we explained the top‐ranked MACCS molecular fingerprints, as shown in Table S9.

**TABLE 1:** Molecular fingerprint and its smart code, and remark have the greatest influence on model performance.

ID	SMART code	Remark
MACCS 133	(‘*@*!@[#7]’, 0)	A$A!N
MACCS 129	('[$(*∼[CH2]∼*∼*∼[CH2]∼*), $([R]1@[CH2;R]@[R]@[R]@[R]@[CH2;R]1), $([R]1@[CH2]@[R]@[R]@[CH2;R]1), $(*∼[CH2]∼[R]1@[R]@[CH2;R]1)]',0)	ACH2AACH2A
MACCS 124	(‘[!#6;!#1]∼[!#6;!#1]’, 0)	QQ
MACCS 88	(‘[#16]’, 0)	S
MACCS 156	(‘[#7]∼*(∼*)∼*’, 0)	NA(A)A
MACCS 160	(‘[C;H3,H4]’, 0)	CH3
MACCS 91	('[$([!#6;!#1;!H0]∼*∼*∼*∼[CH2]∼*), $([!#6;!#1;!H0;R]1@[R]@[R]@[R]@[CH2;R]1), $([!#6;!#1;!H0]∼[R]1@[R]@[R]@[CH2;R]1), $([!#6;!#1;!H0]∼*∼[R]1@[R]@[CH2;R]1)]',0)	QHAAACH2A

**FIGURE 4:**
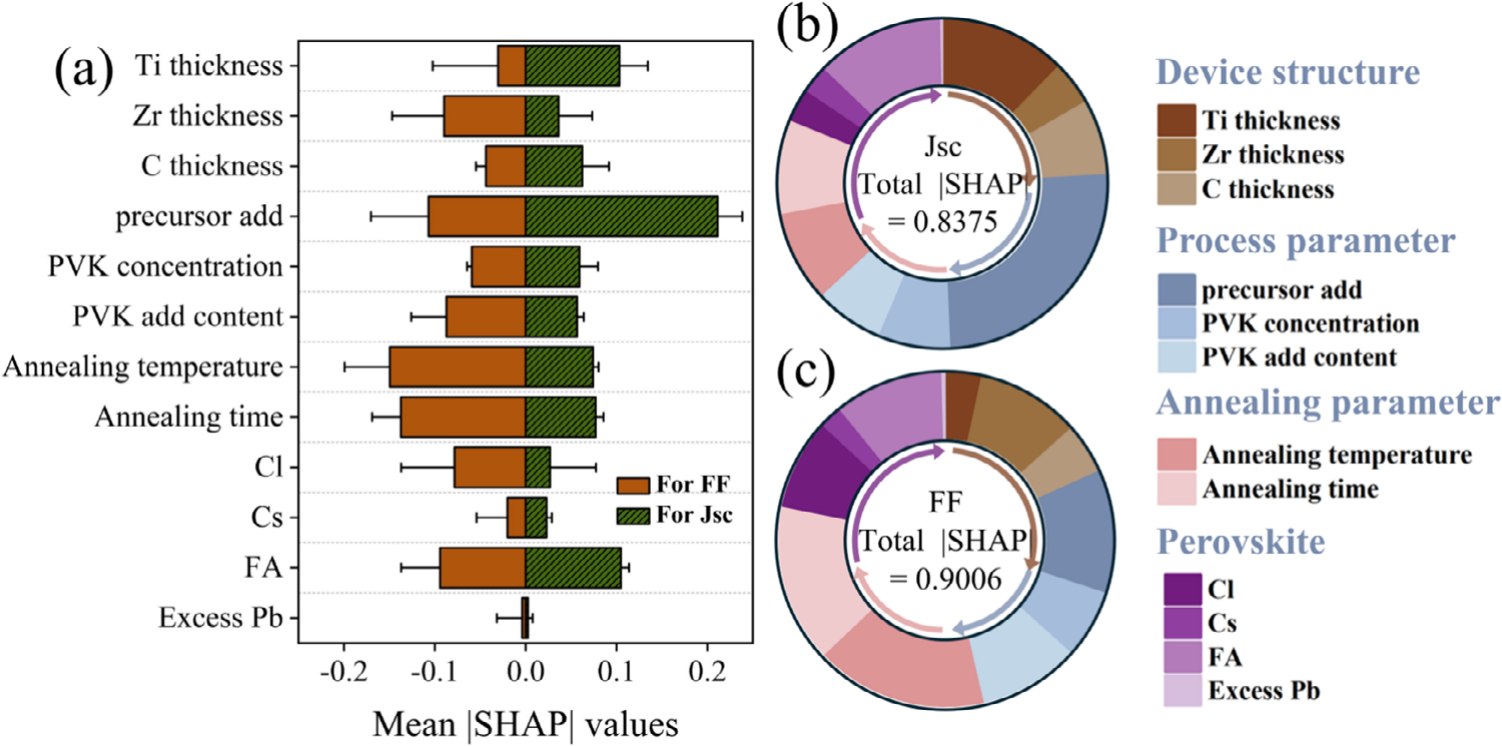
(a) The characteristic importance of each parameter affecting *J*_sc_ and FF, and the distribution comparison of the influence of four parameters on (b) *J*_sc_ and (c) FF of the devices.

To facilitate experimental optimization of device efficiency, the features were reconstructed and categorized into four aspects: device structure, process parameters, annealing parameters, and perovskite composition. The feature importance of *J*_sc_ and FF, which were of greater experimental interest, were analyzed, and the results are presented in Figure 4a. Meanwhile, the characteristic importance of different features for *J*_sc_ and FF is also presented in Figure 4b,c. The results show that the FF is more sensitive to the annealing parameters and perovskite composition, and the device structure and process parameters have a greater influence on the *J*_sc_. It can be seen that the device structure, especially the thickness of the Ti layer, has a significant impact on the *J*_sc_. The mp‐TiO_2_ serves as the electron transport layer. Under light illumination, the perovskite filled in the mp‐TiO_2_ absorbs photons, resulting in the generation of electron‐hole pairs. The photoexcited electrons are then efficiently extracted by the mp‐TiO_2_ and transported to the FTO electrode. The large specific surface area of the mp‐TiO_2_ plays a crucial role in this process, significantly enhancing the rapid and efficient extraction of photoexcited electrons [45]. Meanwhile, as a key factor influencing the bandgap, the selection of perovskite types has demonstrated an impact on *J*_sc_, as the perovskite bandgap can affect the light absorption range of the device. The selection of precursor solution has a significant impact on the filling effect of perovskite and also intensifies its influence on the *J*_sc_. The solvent annealing process and evaporation rate play a key role in the crystallization of perovskite films. By precisely controlling the annealing temperature and solvent evaporation rate, the nucleation and crystal growth of perovskite can be regulated, which directly affects the grain size and morphology of the resulting film [46, 47, 48, 49]. Well‐shaped grains usually lead to better charge transfer and fewer defects, thereby enhancing the FF of PSCs. Current studies predominantly emphasize the selection of annealing parameters, the extension of annealing time, and the utilization of the solvent evaporation‐controlled crystallization (SECC) technique for device annealing. These approaches have been validated as effective strategies to enhance the FF.

Notably, parameter prioritization shows differential dependencies: optimization of device architecture parameters demonstrates effectiveness for *J*_sc_ enhancement, while modification of the perovskite composition, specifically through strategic incorporation of precursor additives, emerges as the most impactful approach for *J*_sc_ improvement. This compositional engineering strategy achieves a 12.3% relative increase in *J*_sc_ compared to baseline configurations, as evidenced by our experimental validation series Figure S12.

### 2.3 Potential Improvement Solutions

**FIGURE 5:**
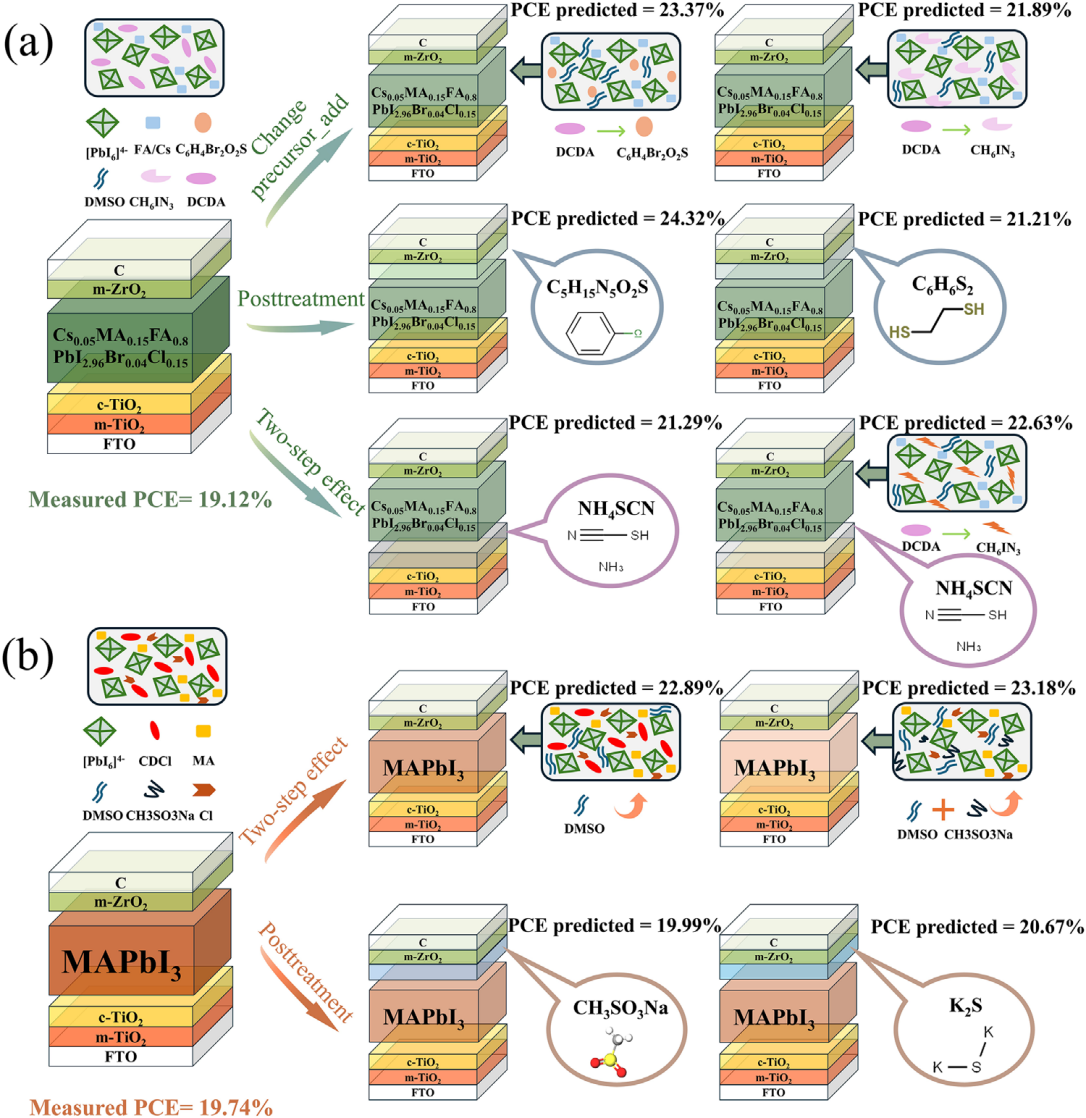
ML solutions for the PCE enhancement of devices with different solutions: (a) DMF/DMSO (4:1) of Zhang et al. [50], and (b) NMF of Han et al. [51].

For the high efficiency strategies given by ML, we selected eight groups for experimental verification (displayed in Table S5), and the maximum efficiency deviation is less than 2.16%, showing the good generalization performance of the ML model. To further improve the guiding role of the ML model in the experiment, we selected the data of the latest published articles as an example. Two mainstream precursor solvent systems were selected for the prediction of high‐efficiency strategies. Following the established rules, the ML model proposed specific strategies for efficiency improvement, the optimization flowchart is shown in Figure S13. The device with DMF/DMSO mixed solution is optimized as shown in Figure 5a, while that with NMF solution is shown in Figure 5b. Through targeted modifications, the stacking model provided actionable insights, including additive adjustments for post‐treatment for Zhang et al.’s device [50], and precursor solvent doping for Han et al.’s device [51]. The stacking model also predicted the PCE values resulting from these optimizations, with the highest predicted PCE reaching an impressive 24.32%. This represents a significant contribution toward breaking the efficiency limitations of p‐MPSCs.

## 3 Conclusions

In this study, we demonstrate the significant potential of ML in advancing the development of p‐MPSCs. By utilizing a comprehensive dataset of 1087 p‐MPSCs, we successfully trained a highly effective efficiency prediction model, achieving an *R* ^2^ of 0.73 and an RMSE of 1.75%. SHAP‐based ML interpretation revealed the optimal range for perovskite components and device processing strategy with considerable potential. Additionally, utilizing RDKit, we identified ten MACCS molecular fingerprints that exert the greatest influence on additive performance, establishing selection rules for additives in p‐MPSCs. The experimental devices guided by our ML model achieved a final efficiency of 19.36%, confirming the reliability of our ML‐driven approach in facilitating breakthroughs in p‐MPSCs efficiency. Furthermore, the model predicts a maximum achievable efficiency of 24.32%, providing a clear direction for future research and highlighting the potential of ML‐driven optimization to substantially improve the efficiency of p‐MPSCs. Overall, this work establishes an integrated ML‐driven framework for p‐MPSCs, combining predictive modeling, molecular fingerprint analysis, and experimental validation to accelerate the development of high‐performance p‐MPSCs devices.

## Author Contributions

Conceptualization: H.M., J.Z., X.Z., Y.W., C.Z., A.Y., W.K., and X.L. Experiments: H.M., X.Z., and M.D. Manuscript writing: X.L., H.M., J.Z., J.L., and M.D. Coordination: X.L., J.Z., and J.L.

## Conflicts of Interest

The authors declare no conflicts of interest.

## Supporting information




**Supporting File**: advs76258‐sup‐0001‐SuppMat.pdf.

## Data Availability

The data that support the findings of this study are available on request from the corresponding author upon reasonable request.
