# Supplementary material for: Advancing the Design of High‐Efficiency Printable Hole‐Conductor‐Free Mesoscopic Perovskite Solar Cells Through Machine Learning
Source: Adv Sci (Weinh). 2026 Jun 22:e76258. Online ahead of print. doi: 10.1002/advs.76258 (PMC13336852; doi:10.1002/advs.76258)
Supplement: Supplementary file 1 — Supporting File: advs76258‐sup‐0001‐SuppMat.pdf. [file ADVS-9999-e76258-s001.pdf]

Supporting Information

Advancing the Design of High-Efficiency Printable Hole-Conductor-Free Mesoscopic Perovskite Solar Cells through Machine Learning

Hao Meng[a#], Jingzi Zhang[a,b#], Xu Zhu[a], Yuelin Wang[a], Antai Yang[a], Kailong Hu[a], Chengquan Zhong[c*], Jiakai Liu[d,e], Menghan Dun[c*] and Xi Lin[a*]*

[a]*School of Materials Science and Engineering, Harbin Institute of Technology, Shenzhen 518055, China*

[b]*Research Institute of Physical Sciences in Special Environments, Harbin Institute of Technology, Shenzhen 518055, China*

[c]*College of Marine Equipment and Mechanical Engineering, Jimei University, Xiamen 361021, Fujian, China*

[d]*Xinjiang Key Laboratory of Separation Material and Technology, Xinjiang Technical Institute of Physics and Chemistry, Chinese Academy of Sciences, Urumqi 830011, China*

[e]*Center of Materials Science and Opto-electronic Technology, University of Chinese Academy of Sciences, Beijing 100049, China*

Corresponding authors

*E-mail: linxi@hit.edu.cn

*E-mail: dunmenghan@jmu.edu.cn

*E-mail: zjzhang@hit.edu.cn

#These authors contributed equally to this work.

Dataset preparation

The dataset consists of 841 data points from articles published between 2019 and 2024 and 237 data points from our experiment, with a PCE range of 0.4 % to 22.2 %. The database contains the device structure, fabrication process, test data, and precursor addition-related feature descriptors of printable mesoscale perovskite solar cells (p-MPSC). The journal literature data is collected from 120 kinds of literature through comprehensive retrieval by keywords “printing”, “Perovskite”, and “mesoporous”.

After a series of strict cleaning rules, the data points were further limited to 986 data points. Specifically, the data set is cleaned according to the following rules: (1) Eliminate data points measured under non-standard test conditions. (2) After removing data points unrelated to the research focus, only the data of p-MPSC were retained. (3) Exclude one or more data points with incomplete input feature information. (4) Adjust the stable PCE value to retain only the highest recorded value of PSCs in the data set. Among them, the higher value of PCE is the backward scanning curve, with a scanning rate of 50 mV/s. (5) Re-examine the composition of the chemical formula and replace the characteristic component with the corresponding proportion of precursor ions. (6) If a data point has the same input characteristics but exhibits a different report PCE, the average PCE is exported and saved as a new data point. The calculation takes into account the experimental environment and the measurement differences inherent in different laboratories.

Dataset process

We carried out the missing value processing work on the dataset and obtained the complete dataset. The specific handling rules are as follows: (1) Data missing of the effective area: The missing values are assigned the mode of the effective area of small-area p-MPSCs devices, which is 0.1 cm^2 if the large-area p-MPSCs device is not explicitly mentioned in the paper, or if the *J-V* curve does not reflect the expected performance of a large-area device. For datasets that explicitly describe the use of large-area devices or indicate that the device was fabricated on 10×10 cm conductive glass, the effective area is set to the mode of the 10×10 cm device, which corresponds to 56.4 cm^2. For all remaining datasets where the device size is not specified, the data have been excluded from the analysis. (2) Data missing of the functional layer thickness: we estimate the thickness of the functional layer through the image scale and fill in the missing values if the data article contains corresponding SEM

cross-sectional images. At the same time, in some cases, we completed the corresponding missing values based on the thickness of the functional layer reported in the author's previous research. For the remaining missing values, we use the mode of the corresponding functional layer thickness to determine the different missing values, that is, to supplement the thicknesses of mesoporous titanium oxide, mesoporous zirconia and mesoporous carbon electrodes to 700 nm, 2 μm and 15 μm respectively. (3) Data missing of the FF and J_{sc}: There are a few cases where the corresponding efficiency is provided in the article but the expressions corresponding to FF and J_{sc} are missing. We retained the corresponding PCE data, but in the subsequent FF and J_{sc} prediction tasks, we deleted the data corresponding to the missing values to maintain the reliability of the model. (4) Data missing for the other features: For the remaining small portion of missing values, we always follow the following principles. For a small proportion of missing values, interpolation processing should be carried out according to the actual situation (such as mean, mode, regression interpolation, etc.). For a large proportion of missing values, to prevent the introduction of bias, we should use interpolation processing with caution. For some extreme features, deletion processing can be considered.

We simultaneously standardized the data, converting it to a common scale to facilitate comparison and modeling by the model.

Feature generation

Categorical feature encoding constitutes a critical preprocessing step for machine learning applications in perovskite photovoltaic studies. The label encoding technique facilitates the transformation of discrete categorical variables (e.g., solvent types or annealing conditions) into sequentially indexed integers through injective mapping, thereby establishing numerical representations while preserving categorical cardinality. In contrast, one-hot encoding generates orthogonal binary vectors through Kronecker delta expansion, effectively eliminating potential artificial ordinal relationships between categories that might bias machine learning models.

These encoding methodologies enable the representation of experimental categorical parameters (such as precursor compositions or device architectures) in Euclidean vector spaces, fulfilling the prerequisite of numerical input formats for regression-based predictive modeling. The implementation of appropriate encoding strategies ensures optimal algorithm compatibility with conventional machine

learning frameworks while maintaining critical information integrity throughout the feature engineering pipeline.

The 8 ionic ratio features of MA^+ (methylammonium), FA^+ (formamidinium), Cs^+, Rb^+, Pb^{2+}, Sn^{2+}, Br^-, and I^- were extracted from the perovskite components factor. The bandgap feature was predicted using the model developed by Gok et al, with the performance shown in **Figure S1**. The remaining 15 process-related factors (ETL, Spacer, Functional layer, perovskite components, precursor_solution, deposition procedure, deposition method, solution_add, precursor_add, type, pretreatment, Annealing, thickness, add-Cl, add-Pb) were encoded by a label encoding method by integers starting from 0. Some features that are considered important, such as "SECC", “5-avai”, “DMF/ DMSO4:1”, etc., are constructed by one-hot encoding. Finally, 30 features listed in **Table S2** were generated through the feature engineering process.

Model selection

Linear Regression (LR) demonstrates notable efficacy in low-dimensional datasets with linear correlations, benefiting from its mathematical transparency and computational efficiency. The parametric approach enables rapid model construction through closed-form solutions, particularly suitable for preliminary analysis of photovoltaic process parameters.

Random Forest (RF) enhances predictive stability through bootstrap aggregating of decorrelated decision trees. The embedded feature subspace sampling mechanism naturally accommodates categorical experimental parameters (e.g., solvent types) and mitigates measurement uncertainties in high-throughput photovoltaic testing.

LightGBM (LGBM) accelerates gradient boosting through histogram-based approximation and exclusive feature bundling. The leaf-wise growth strategy coupled with vertical optimization demonstrates advantages in processing spectral data and large-scale device performance databases.

XGBoost incorporates second-order Taylor expansion in loss function optimization, integrating sparsity-aware split finding with regularization constraints. The column block architecture enables efficient handling of mixed-type features prevalent in perovskite compositional engineering.

CatBoost implements ordered boosting with permutation-driven gradient estimation to prevent target leakage. The oblivious tree structure combined with categorical feature processing algorithms provides reliable performance in small-sample scenarios of novel photovoltaic material exploration.

Gradient Boosting Machine (GBM) employs iterative functional gradient descent to construct additive models through stage-wise optimization. By sequentially fitting weak learners to residual errors using differentiable loss functions (e.g., squared error for regression), this method achieves progressive refinement of photovoltaic performance predictions. The shrinkage regularization technique embedded in the learning rate parameter effectively balances bias-variance tradeoffs during perovskite material screening processes.

AdaBoost (Adaptive Boosting) implements an exponential loss minimization framework via adaptive sample re-weighting. The algorithm dynamically increases emphasis on misclassified device characterization data points through multiplicative weight updates, while maintaining compatibility with heterogeneous experimental parameters in thin-film process optimization. This weighted majority voting mechanism demonstrates efficacy in stability assessment tasks for perovskite solar cells under varying environmental stressors.

Ridge Regression addresses multicollinearity in photovoltaic parameter optimization through $\ell 2$-norm regularization. By imposing spectral constraint on coefficient magnitudes via Tikhonov matrix conditioning, this method stabilizes the inverse operation in ill-posed scenarios typical of perovskite compositional analysis. The regularization hyperparameter λ mediates bias-variance equilibrium when processing high-dimensional characterization data from combinatorial material libraries.

Support Vector Machine (SVM) constructs maximum-margin hyperplanes in kernel-induced feature spaces, implementing Vapnik-Chervonenkis dimension control through convex quadratic programming. The kernel trick (e.g., Gaussian RBF or polynomial) enables nonlinear separation of device performance clusters in Hilbert space, particularly effective for binary classification tasks in perovskite defect state identification. The soft-margin extension with slack variables accommodates experimental noise inherent in optoelectronic characterization datasets.

Model evaluation

The correlation coefficient (r) serves as a statistical metric to quantify the strength of linear relationships between predicted and observed values, offering insights into model fit quality and prediction reliability. Its value ranges from -1 to 1, with proximity to ±1 signifying higher predictive precision:

$r = 1$: Perfect positive correlation (aligned variation).

r = -1: Perfect negative correlation (inverse variation).

r = 0: Absence of linear association.

The coefficient of determination (R^2) measures the proportion of variance in the dependent variable explained by the model, serving as a key indicator of goodness-of-fit. It spans [0, 1], where values approaching 1 denote superior model performance in capturing data patterns and explaining variability.

The root means square error (RMSE) evaluates prediction accuracy by amplifying the impact of larger errors through squaring, thereby reflecting the overall dispersion of prediction deviations. Lower RMSE values indicate tighter alignment between predictions and ground-truth observations.

The mean absolute error (MAE) calculates the average magnitude of absolute prediction errors. By assigning equal weight to all deviations, it provides an intuitive, outlier-resistant measure of error magnitude while maintaining computational simplicity.

The calculation formulas are given below. Where $\hat{y}_i$ is the predicted value of the model, y_i is the true value, $\bar{y}$ is the average value, and $\bar{\hat{y}}$ represents the average predicted value.

$$r=\frac{\sum_{i=1}^{n}(y_i-\bar{y})\left(\hat{y}_i-\bar{\hat{y}}\right)}{\sqrt{\sum_{i=1}^{n}(y_i-\bar{y})^2\sum_{i=1}^{n}\left(\hat{y}_i-\bar{\hat{y}}\right)^2}}$$

$$R^2=1-\frac{\sum_{i=1}^{n}(y_i-\hat{y}_i)^2}{\sum_{i=1}^{n}(y_i-\bar{y})^2}$$

$$RMSE=\sqrt{\frac{1}{n}\sum_{i=1}^{n}(y_i-\hat{y}_i)^2}$$

$$MAE=\frac{1}{n}\sum_{i=1}^{n}|\hat{y}_i-y_i|$$

Materials and preparation

Formamidinium iodide (FAI) and lead iodide (PbI_2) were purchased from Aladdin reagent. Cesium iodide (CsI) and Methylamine iodide (MAI) were purchased

from TCI. N-Methyl formamide (NMF), N, N-dimethylformamide (DMF) and dimethyl sulfoxide (DMSO) were purchased from Aladdin reagent. Isopropanol (IPA) was purchased from Aladdin reagent, titanium diisopropoxide bis(acetylacetonate) was purchased from Sigma–Aldrich. TiO_2 paste (30NR-D) was purchased from Great Cell. ZrO_2 paste and carbon paste were purchased from Wonder Solar Co., Ltd. Unless otherwise stated, all the materials were purchased and used directly without further purification.

Paste and Precursor Preparation: The TiO_2 paste used was obtained from 30NR-D paste dispersed in turpentine at a weight ratio of 1:4. The perovskite precursor was prepared as required by experimental verification.

Device fabrication

The glass/FTO substrates were ultrasonically cleaned sequentially with soapy water, deionized water, and ethanol for 30 min, respectively. Then, the dried FTO substrates were treated with ultraviolet ozone (UVO) for 20 min. The compact TiO_2 (c-TiO_2) layer was deposited on the FTO substrate by spray pyrolysis at 450 °C with titanium diisopropoxide bis(acetylacetonate) solution (3mL in 150 mL anhydrous ethanol) and maintained at 450 °C for 30 min. Printing three-layer mesoporous film: TiO_2 layer is printed on a dense layer by a screen-printing machine, placed flat on a table and left to stand for 2 hours to spread out. Then, it is dried under a hot table at 75 °C to remove turpentine alcohol, and sintered and kept warm in a hot table at 500 °C. After natural cooling, the mesoporous TiO_2 layer is obtained. The layer of ZrO_2 and layer of C were printed successively on the mesoporous TiO_2 layer in the same way. They were sintered in a hot stage at 400 °C and kept warm. After cooling, a three-layer mesoporous film substrate was obtained. After cooling to room temperature, perovskite precursor solution: MACl (0.0101 g, 0.15 mmol), CsI (0.0130 g, 0.05 mmol), MAI (0.0239 g, 0.15 mmol), FAI (0.1376 g, 0.8 mmol) and PbI_2 (0.4610 g, 1 mmol) and 0.8 mL DMF/DMSO (4:1) with 5 % FA mixed solvent, was filled at the edge of electrode C on a three-layer mesoporous substrate by the one-step drop-coating method, then let it stand for the solution to fully permeate and anneal at 57 °C for 20 hours to remove the solvent. Thus, the fabrication of p-MPSCs is completed. For our optimal devices, they are fabricated in a dust-free room and annealed simultaneously in a glove box (with a stable temperature of 25 °C and humidity maintained at 30 %).

Device characterizations

The device performance was measured by a Keithley 2400 source meter under air mass 1.5 (AM1.5) illumination at 100 mW cm^{-2} with a scan rate of 50 mV s^{-1}. The active area of the device is approximately 0.64 cm^2, and a mask with an oblong aperture (0.12 cm^2) was applied under *J*-*V* tests.

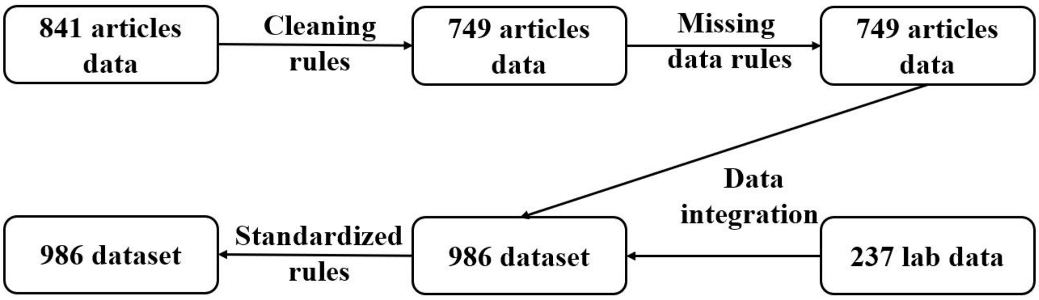

Fig. S1 Database data filtering flowchart.

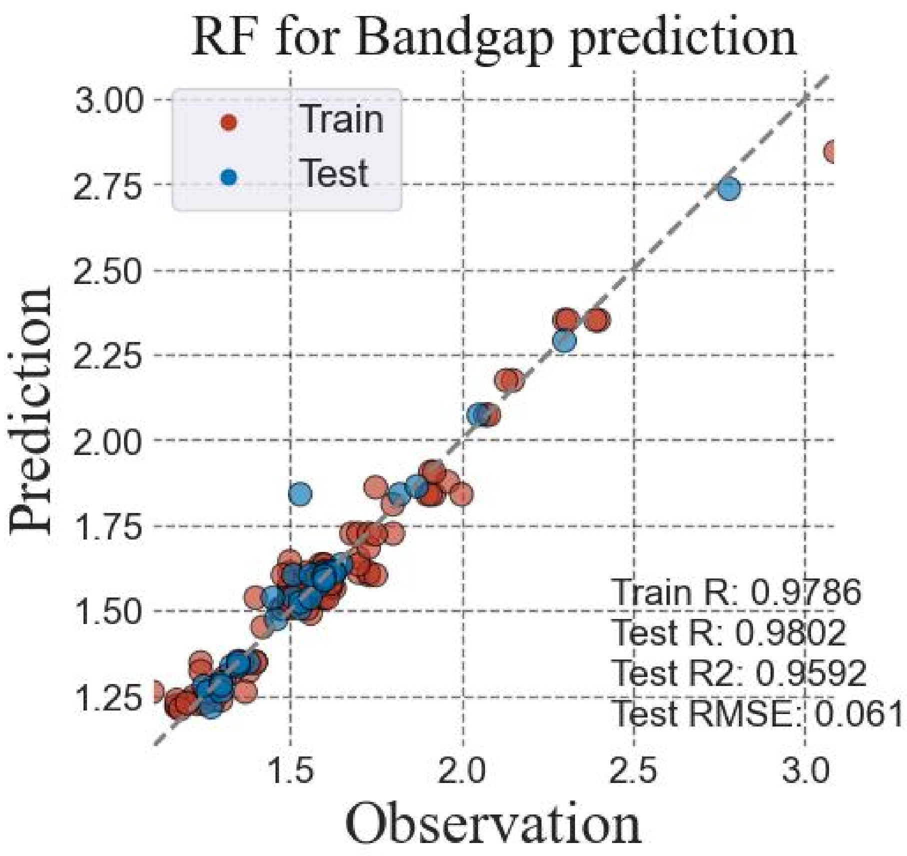

Fig. S2 The prediction effect of bandgaps using RF, with data and methods provided by Gok et al.

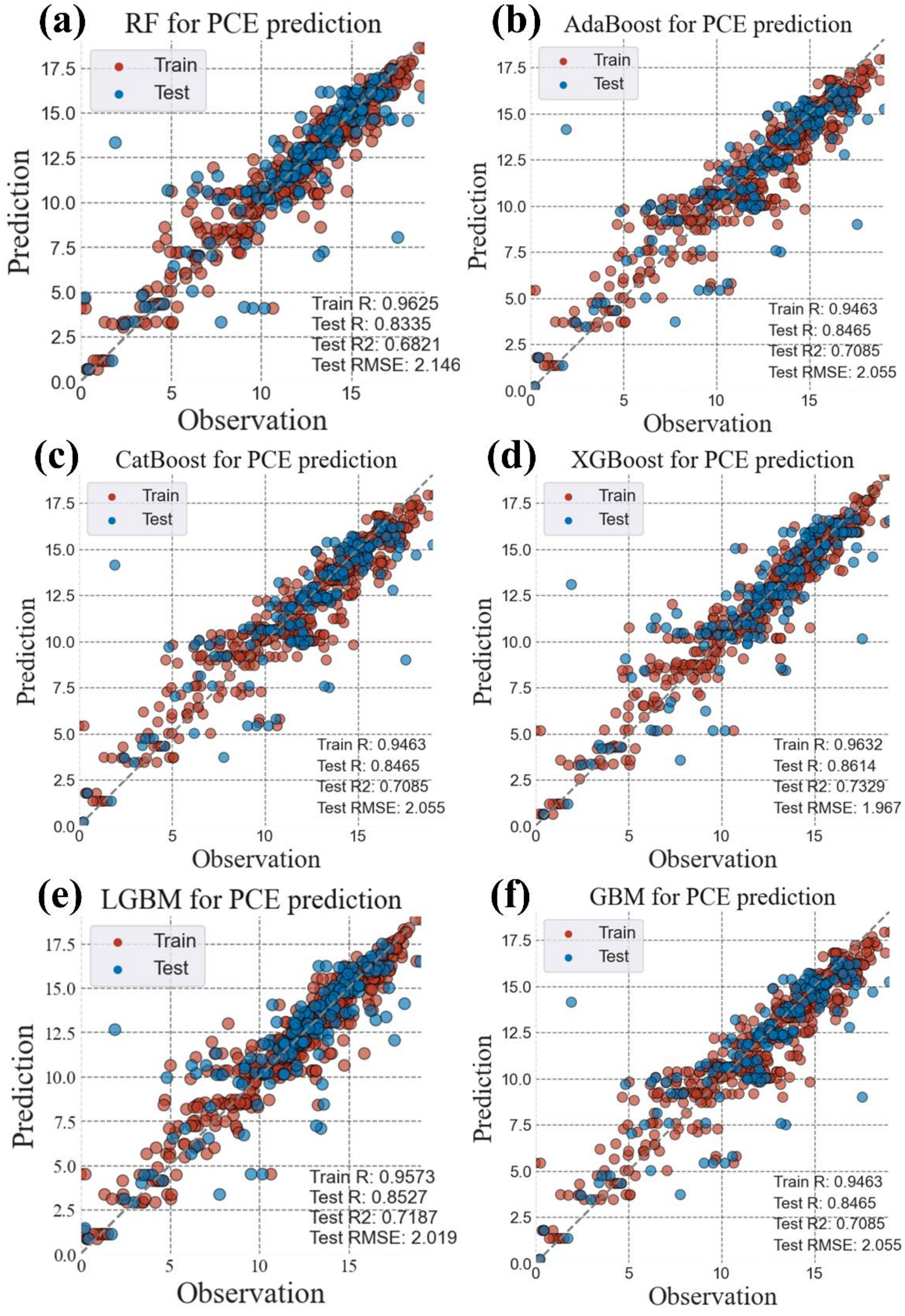

Fig. S3 The fitting graph of PCE results using six different traditional machine learning models for PCE prediction, where red represents the training set and blue represents the test set: (a) Random Forest (RF), (b) Adaptive Boosting (AdaBoost), (c) Categorical Boosting (CatBoost), (d) Extreme Gradient Boosting (XGBoost), (e) Light Gradient Boosting Machine (LGBM), (f) Gradient Boosting Machine (GBM).

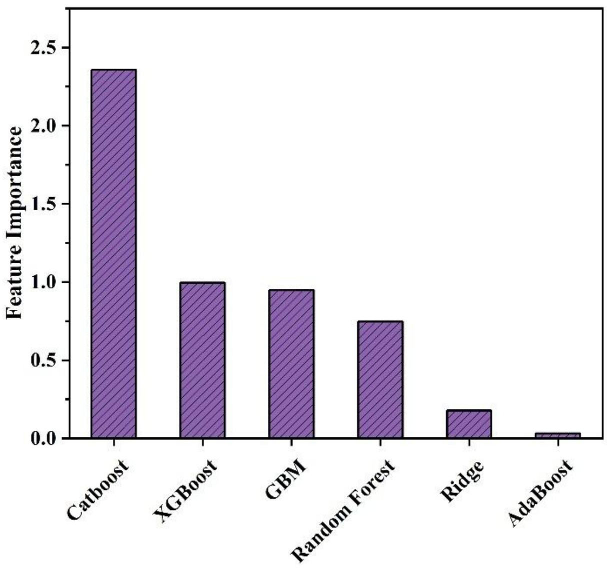

Fig. S4 Rank the importance of the contribution features of each base model in the stacking model to the stacking integrated model.

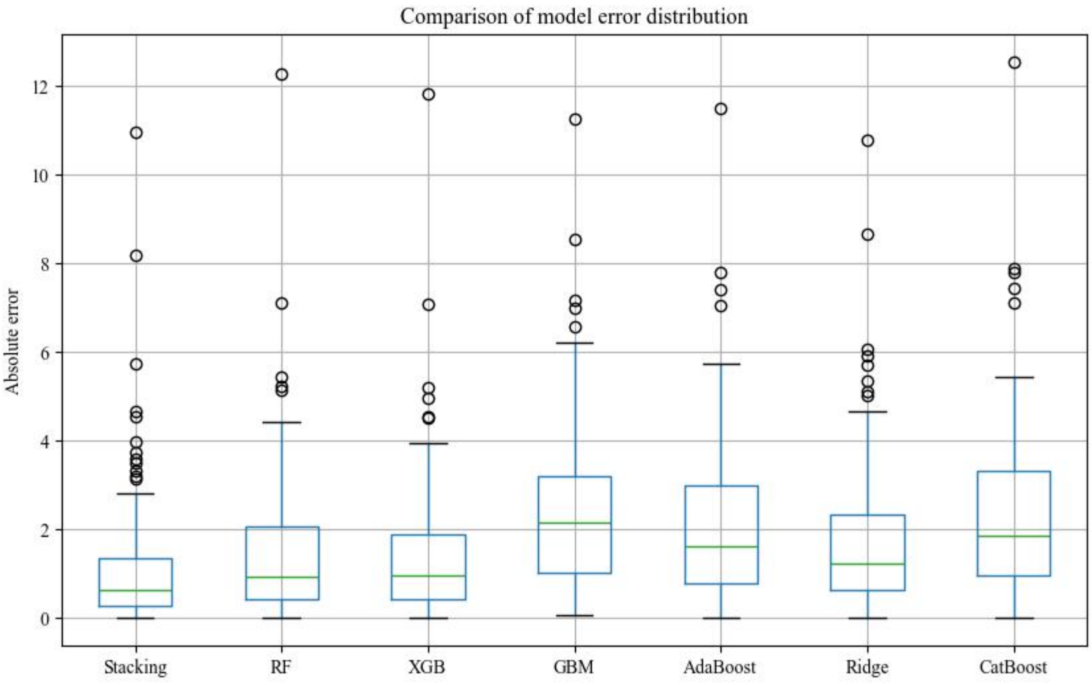

Fig. S5 Comparison of the absolute error between the stacking model and the other models.

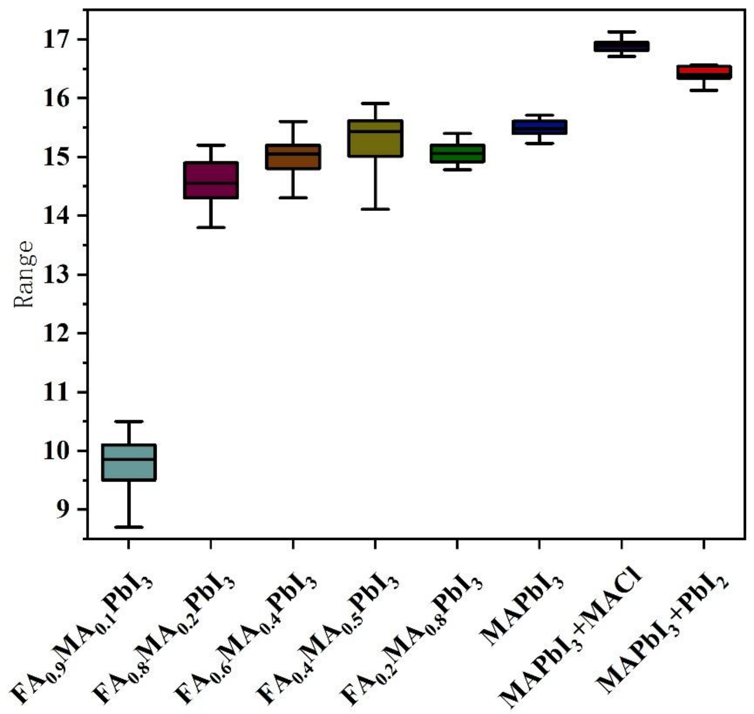

Fig. S6 Statistics of the PCE of p-MPSCs with different groups based on 8 devices. The central line represents the median, the box limits correspond to the upper and lower quartiles, and the whiskers extend to the minimum and maximum values.

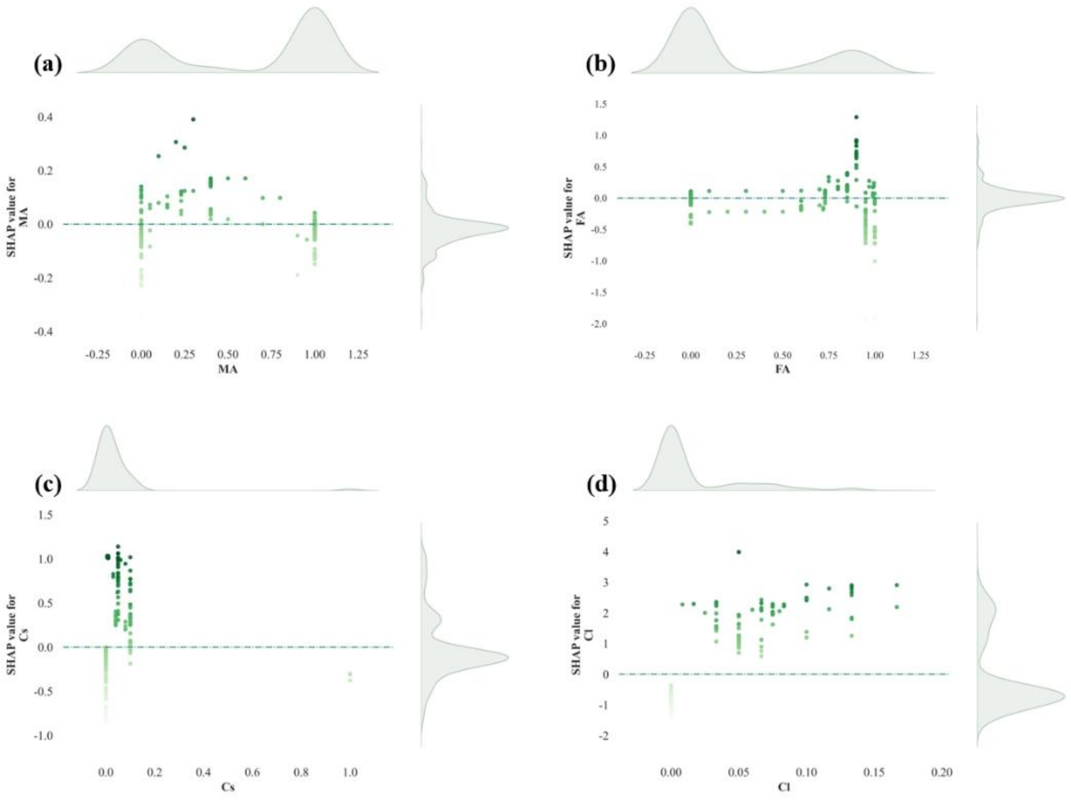

Fig. S7 Contributions of different ion ratios to PCE. The horizontal axis in the figure represents the content ratio of different ions, while the vertical axis represents their SHAP values, which represents the contribution to PCE of each data point in the dataset.

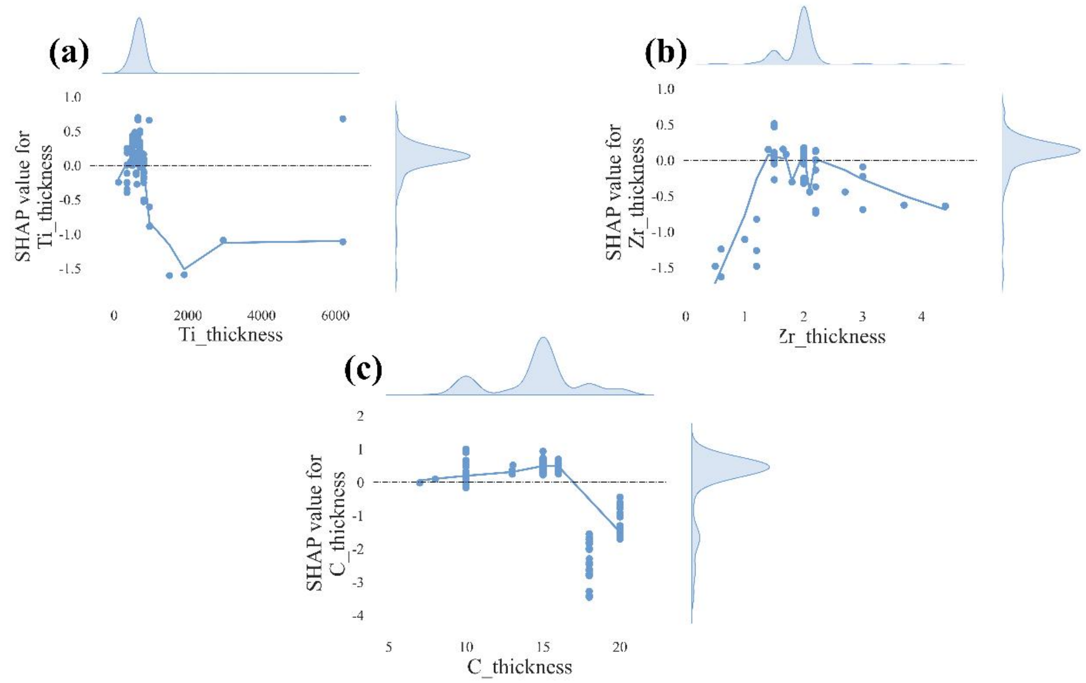

Fig. S8 Contributions of the thickness of each layer of the device structure to PCE. The horizontal axis in the figure represents the content ratio of different ions, while the vertical axis represents their SHAP values, which represents the contribution to PCE of each data point in the dataset.

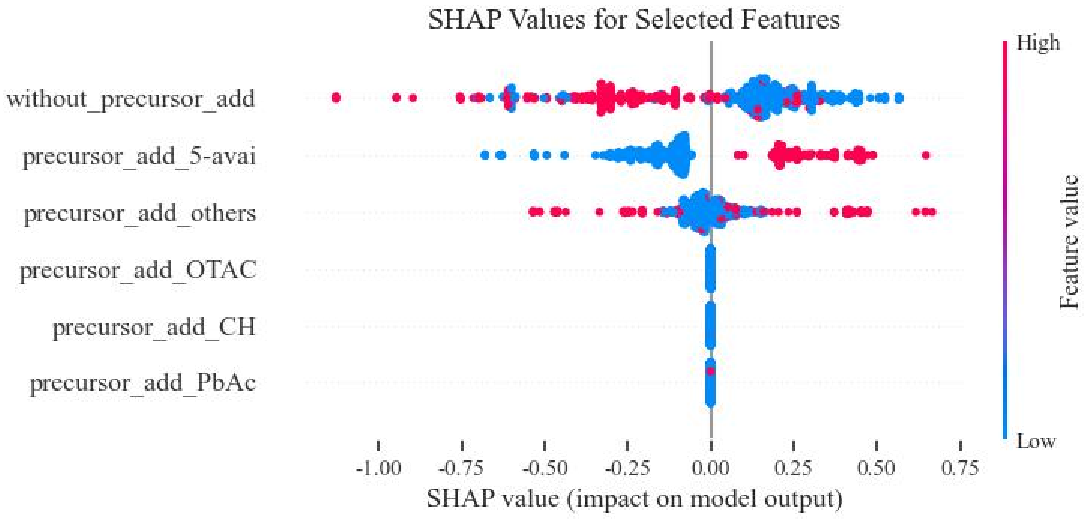

Fig. S9 The ranked importance of anti-solvent and the impact of alternatives, with a pink background representing positive contribution and a light blue background representing negative contribution. The red dots represent the data using this option,

while the blue dots indicate not using it.

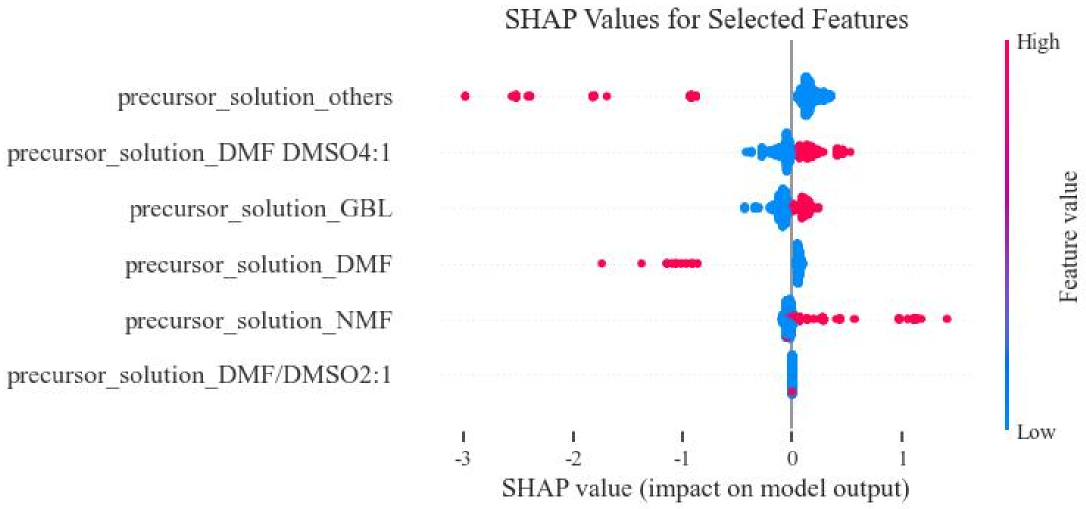

Fig. S10 The ranked importance of ETL and the impact of alternatives, with a pink background representing positive contribution and a light blue background representing negative contribution. The red dots represent the data using this option, while the blue dots indicate not using it.

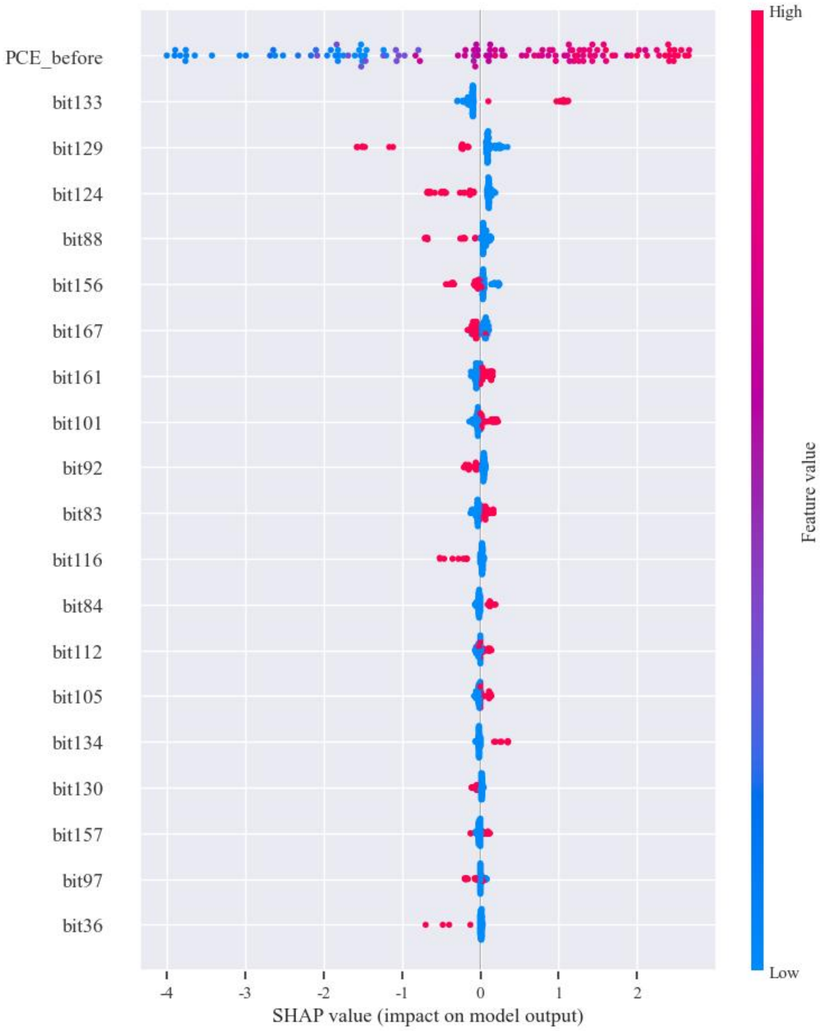

Fig. S11 The ranked importance of the precursor additive molecular fingerprint. The red dots represent the data using this option, while the blue dots indicate not using it.

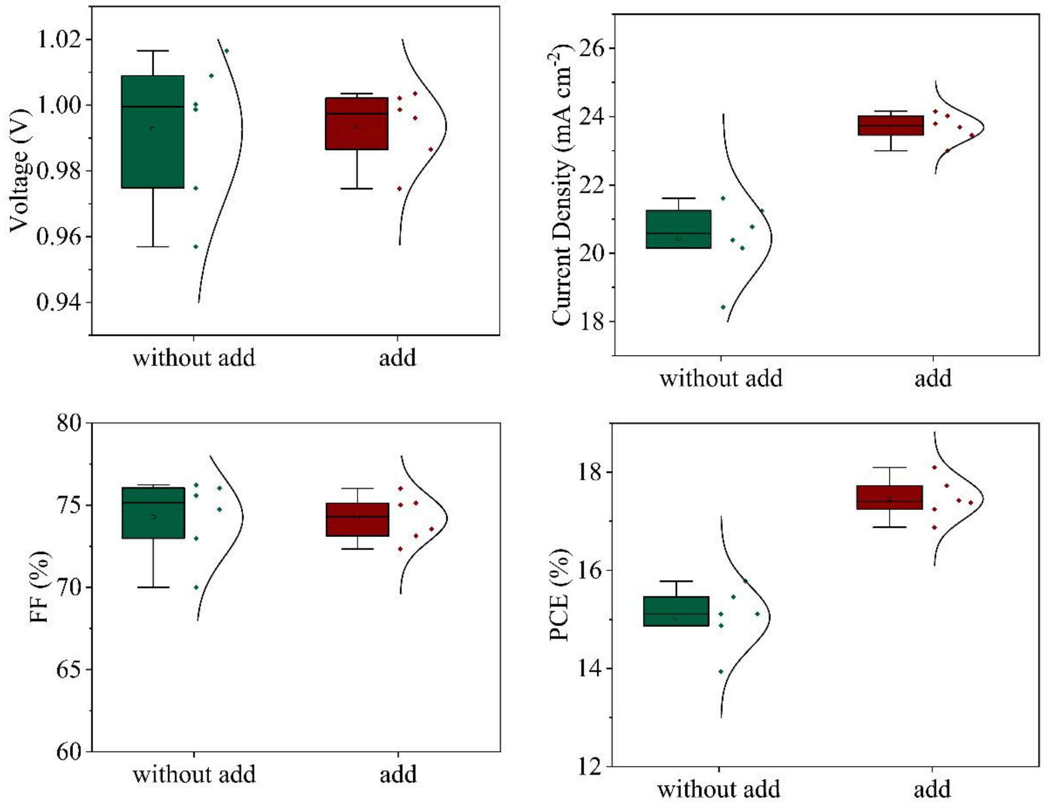

Fig. S12 Statistical performance parameters of the devices with or without precursor add (6 devices were used per group).

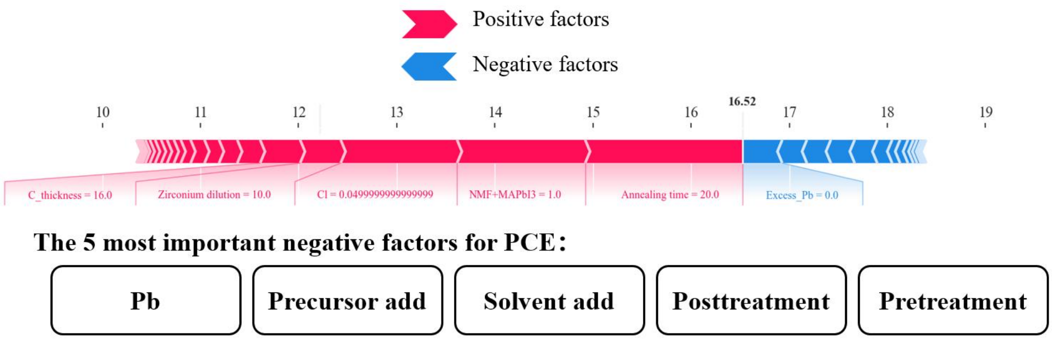

Fig. S13 SHAP force-plot–assisted schematic for guiding optimization directions.

Table S1. Performance development of p-MPSCs.

Year	Device structure	V_{oc}	J_{sc}	FF	PCE	Ref
2013	FTO/c-TiO_2/mp-TiO_2/ mp ZrO_2/Carbon	0.88	10.6	0.61	6.6	*Sci Rep*. 3, 3132 (2013)
2014	FTO/c-TiO_2/mp-TiO_2/ mp ZrO_2/Carbon	0.86	22.8	0.66	12.8	*Science* 345, 295 (2014)
2015	FTO/c-TiO_2/mp-TiO_2/ mp ZrO_2/Carbon	0.87	22.9	0.67	13.4	*J. Mater. Chem. A*. 3, 9103 (2015)
2016	FTO/c-TiO_2/mp-TiO_2/ mp ZrO_2/Carbon	0.93	20.2	0.77	14.5	*J. Mater. Chem. A*, 4, 16731 (2016)
2017	FTO/c-TiO_2/mp-TiO_2/ mp ZrO_2/Carbon	0.94	21.5	0.77	15.6	*Nat Commun*. 8, 14555 (2017).
2018	FTO/c-TiO_2/mp-TiO_2/ mp ZrO_2/Carbon	0.98	23.2	0.69	15.7	*Nano Energy* 53,160 (2018).
2020	FTO/c-TiO_2/mp-TiO_2/ mp ZrO_2/Carbon	1.02	23.1	0.70	16.5	*Sol. RRL* 4: 2000185 (2020).
2021	FTO/c-TiO_2/mp-TiO_2/ mp ZrO_2/Carbon	1.01	24.5	0.73	18.1	*Fundamental Research* 1, 385 (2021)
2022	FTO/c-TiO_2/mp-TiO_2/ mp ZrO_2/Carbon	1.03	22.8	0.80	18.8	*Fundamental Research* 2, 276 (2022)
2023	FTO/c-TiO_2/mp-TiO_2/ mp ZrO_2/Carbon	1.01	24.2	0.78	19.0	*Small*, 2307246 (2023)
2024	FTO/c-TiO_2/mp-TiO_2/ mp ZrO_2/Carbon	1.06	25.6	0.82	22.2	*Science*, 383, 1198 (2024)
2025	FTO/c-TiO_2/mp-TiO_2/ mp ZrO_2/Carbon	1.11	25.3	0.83	23.2	*Nat Energy* 10, 1084–1094 (2025)

Table S2. The features encoding method and description for 30 features in the dataset.

Num	Feature name and encoding method	Feature description
1	MA ratio (extracted from perovskite components)	Continuous numbers from 0 to 1
2	FA ratio (extracted from perovskite components)	Continuous numbers from 0 to 1
3	Cs ratio (extracted from perovskite components)	Continuous numbers from 0 to 1
4	Rb ratio (extracted from perovskite components)	Continuous numbers from 0 to 1
5	Pb ratio (extracted from perovskite components)	Continuous numbers from 0 to 1
6	Sn ratio (extracted from perovskite components)	Continuous numbers from 0 to 1
7	Br ratio (extracted from perovskite components)	Continuous numbers from 0 to 1
8	I ratio (extracted from perovskite components)	Continuous numbers from 0 to 1
9	Bandgap (predicted from perovskite components by Gok et al.'s model)	Continuous numbers
10	ETL (encoded by label-encoder)	Contains 3 alternatives
11	Spacer (encoded by label-encoder)	Contains 2 alternatives
12	Functional layer (encoded by label-encoder)	Contains 4 alternatives

13	Perovskite_deposition_procedure (encoded by label-encoder)	Contains 4 alternatives
14	Perovskite_deposition_method (encoded by label-encoder)	Contains 2 alternatives
15	Additives (encoded by label-encoder)	Contains 59 alternatives
16	Precursor_solution (encoded by label-encoder)	Contains 18 alternatives
17	Solvent add (encoded by label-encoder)	Contains 6 alternatives
18	Postprocessing (encoded by label-encoder)	Contains 24 alternatives
19	Environment (encoded by label-encoder)	Contains 3 alternatives
20	C_thickness (encoded by label-encoder)	Continuous numbers
21	Zr_thickness (encoded by label-encoder)	Continuous numbers
22	Ti_thickness (encoded by label-encoder)	Continuous numbers
23	Effective_area (encoded by label-encoder)	Continuous numbers
24	Excess_Cl (encoded by label-encoder)	Contains 2 alternatives
25	Excess_Pb (encoded by label-encoder)	Contains 2 alternatives
26	SECC (encoded by label-encoder)	Contains 2 alternatives

27	5-avai (encoded by label-encoder)	Contains 2 alternatives
28	DMF/DMSO4:1 (encoded by label-encoder)	Contains 2 alternatives
29	NMF+$MAPbI_3$ (encoded by label-encoder)	Contains 2 alternatives
30	GBL+$MAPbI_3$ (encoded by label-encoder)	Contains 2 alternatives

Table S3. Different ML algorithms' best parameters in PCE prediction tasks.

ML algorithms	Best parameters
Ridge	/
RF	'min_samples_leaf': 1,<br>'min_samples_split': 2, 'n_estimators': 60
GBM	' max_depth ': 3, ' min_samples_split': 2,<br>'learning_rate': '0.2',<br>'n_estimators ': '200', 'subsample': '1.0'
CatBoost	'depth': 7,<br>'iterations': 200, 'learning_rate': 0.2
XGBoost	'n_estimators': 500,<br>'learning_rate': 0.05,<br>'max_depth': 5
LGBM	'n_estimators': 800,<br>'learning_rate': 0.1,<br>'num_leaves': 15,<br>'max_depth': 10,
AdaBoost	'learning rate': 0.01, 'max depth': 10,

Table S4. Stacking model to compare the parameters of different models: t-stat and p-value.

	RF	XGB	GBM	Ada Boost	Ridge	Cat Boost	Variance
**t-stat**	-3.73	-3.63	-9.87	-8.32	-5.98	-9.24	13.32
**p-value**	$2.70e^{-04}$	$3.89e^{-04}$	$4.79e^{-18}$	$4.73e^{-14}$	$1.54e^{-08}$	$2.21e^{-16}$	$1.37e^{-14}$

Table S5. Comparison of the PCE prediction task.

Year	Features	Data volume	r	R^2	RMSE(%)
2022	Perovskite family, Device structure, and HTL descriptors	269	0.72	-	3.00
2019	Perovskite composition, and Device descriptors (ΔH, ΔL, Bandgap)	333	0.80	-	3.23
2022	Perovskite composition, and Device descriptors (ΔH, ΔL, Hole mobility, Electron mobility)	248	0.86	-	1.58
2023	Perovskite composition, Material selection, Device structure, Manufacturing methods, Anti-solvent descriptors	1072	0.77	-	1.28
2019	Perovskite family, Material selection, Device structure, Manufacturing methods	1408/515	-	-	3.56/3.38
2021	Perovskite family, Material selection, Device structure, Manufacturing methods	1820	0.66	0.43	3.81
2024	Perovskite composition, Material selection, Device structure, Manufacturing methods	2079	0.87	0.76	2.63
**This work**	**Perovskite composition, Material selection, Device structure, Manufacturing methods**	**1087**	**0.86**	**0.73**	**1.96**

Table S6. Comparison of experimental, prediction interval and predicted PCE values for different groups of devices.

Perovskite	Prediction intervals	Predicted PCE (%)	Experimental verification (%)	Absolute Error (%)
$FA_{0.9}MA_{0.1}PbI_3$	[8.30,10.72]	7.84	10.00	2.16
$FA_{0.8}MA_{0.2}PbI_3$	[12.12,17.39]	16.08	14.90	1.18
$FA_{0.6}MA_{0.4}PbI_3$	[14.99,17.05]	16.15	15.62	0.53
$FA_{0.4}MA_{0.6}PbI_3$	[14.61,15.42]	15.79	15.11	0.68
$FA_{0.2}MA_{0.8}PbI_3$	[14.31,15.49]	15.01	15.27	0.26
$MAPbI_3$	[15.47,16.08]	16.19	15.60	0.59
$MAPbI_3$+MACl	[17.48,19.33]	17.90	16.95	0.95
$MAPbI_3$+ PbI_2	[14.81,17.00]	17.16	16.03	1.13

Table S7. The performance of control devices and target devices.

**Control devices**				**Target devices**			
V_{oc} (V)	J_{sc} (mA cm^{-2})	FF (%)	PCE (%)	V_{oc}(V)	J_{sc} (mA cm^{-2})	FF (%)	PCE (%)
1.01	21.65	76.04	16.63	1.02	24.62	73.89	18.56
1.01	21.89	77.12	17.05	1.00	24.09	74.68	18.08
1.02	22.13	76.55	17.27	1.01	25.23	74.28	19.01
1.02	22.03	74.23	16.68	1.01	24.88	73.21	18.49
1.00	20.68	72.06	14.96	1.01	25.14	75.04	19.04
1.00	21.87	74.89	16.46	1.01	24.68	74.13	18.52

Table S8. The mean ± standard deviation across multiple batches.

p-MPSC Devices		V_{oc} (V)	J_{sc} (mA cm^{-2})	FF (%)	PCE (%)
**Control**	Average	1.01±0.01	21.71±1.0	74.15±3.1	16.51±1.6
	Champion	1.02	22.13	76.55	17.27
**Target**	Average	1.01±0.01	24.73±0.8	75.18±1.0	18.88±0.5
	Champion	1.01	25.32	75.69	19.36

Table S9. MACCS fingerprints and their corresponding explanations.

ID	Explanation
MACCS 133	A nitrogen atom (N) is connected to an atom on a ring system through a non-ring bond (i.e., a chain bond)
MACCS 129	A central non-hydrogen atom is simultaneously connected to a methylene group (-CH_2-) and another non-hydrogen atom, and this methylene group is also connected to another non-hydrogen atom
MACCS 124	Two heteroatoms that are neither hydrogen nor carbon are connected by any type of chemical bond
MACCS 88	Containing any form of sulfur atoms (S)
MACCS 156	A trivalent central atom, one end of which is connected to nitrogen and the other two ends to other groups
MACCS 160	Containing terminal methyl groups (-CH_3) or methane (CH_4)
MACCS 91	A heteroatom is separated from a methylene group (CH_2) by a specific chain or ring structure consisting of approximately 3 to 4 atoms
